# Reduced Rate of Neural Differentiation in the Dentate Gyrus of Adult Dysbindin Null (Sandy) Mouse

**DOI:** 10.1371/journal.pone.0015886

**Published:** 2011-01-18

**Authors:** Naomi Nihonmatsu-Kikuchi, Ryota Hashimoto, Satoko Hattori, Shinsuke Matsuzaki, Takiko Shinozaki, Haruka Miura, Shigeru Ohota, Masaya Tohyama, Masatoshi Takeda, Yoshitaka Tatebayashi

**Affiliations:** 1 Mood Disorders Research Team, Tokyo Institute of Psychiatry, Tokyo, Japan; 2 Graduate School of Biomedical Sciences, Hiroshima University, Hiroshima, Japan; 3 The Osaka-Hamamatsu Joint Research Center for Child Mental Development, Graduate School of Medicine, Osaka University, Osaka, Japan; 4 Department of Psychiatry, Osaka University Graduate School of Medicine, Osaka, Japan; 5 Department of Mental Disorder Research, National Institute of Neuroscience, National Center of Neurology and Psychiatry, Tokyo, Japan; 6 Department of Anatomy and Neuroscience, Graduate School of Medicine, Osaka University, Osaka, Japan; 7 Department of Child Development and Molecular Brain Science, United Graduate School of Child Development, Osaka University, Kanazawa University and Hamamatsu University School of Medicine, Osaka, Japan; RIKEN Brain Science Institute, Japan

## Abstract

Genetic variations in the gene encoding dysbindin has consistently been associated with schizophrenia and bipolar disorder, although little is known about the neural functions carried out by dysbindin. To gain some insight into this area, we took advantage of the readily available dysbindin-null mouse sandy (sdy−/−) and studied hippocampal neurogenesis using thymidine analogue bromodeoxuridine (BrdU). No significant differences were found in the proliferation (4 hours) or survival (1, 4 and 8 weeks after the last BrdU injection) of progenitors in the subgranular regions of the dentate gyrus between sdy−/− and sdy+/+ (control) mice. However, 4 weeks after the last BrdU injection, a significant reduction was observed in the ratio of neuronal differentiation in sdy−/− when compared to that of sdy+/+ (sdy+/+  = 87.0±5.3% *vs.* sdy−/−  = 71.3±8.3%, *p* = 0.01). These findings suggest that dysbindin plays a role during differentiation process in the adult hippocampal neurogenesis and that its deficit may negatively affect neurogenesis-related functions such as cognition and mood.

## Introduction

The dysbindin-1 gene (dystrobrevin-binding protein 1) was originally identified as a gene associated with schizophrenia through its linkage to chromosome 6p [1]. Several subsequent studies have replicated the association between this locus and schizophrenia. In addition, two recent and independent reports have linked certain dysbindin-1 risk haplotypes with bipolar disorder [2], [3]. Dysbindin-1 is widely distributed in the brain, and has been detected both pre- and post-synaptically [4]. A recent immunoelectron microscopy study further revealed that, in the hippocampus, dysbindin-1 is located in synaptic vesicles of axospinous terminals in the dentate gyrus inner molecular layer (DGiml) and CA1 stratum radiatum and in postsynaptic densities and microtubules of dentate hilus neurons and CA1 pyramidal cells [5].

To date, no amino acid sequence mutation in the dysbindin-1 protein that might contribute to the risk of major psychosis has been identified. Furthermore, several studies have implicated the involvement of many different alleles and haplotypes as susceptibility variants. These polymorphisms, however, may modulate dysbindin-1 expression levels since reduced dysbindin message and/or protein levels have been found in schizophrenic brains such as prefrontal cortex and hippocampal formation, brain areas commonly affected by the disorder [6], [7], [8]. In the hippocampus of schizophrenic patients, dysbindin-1 reductions occur in the synaptic terminal fields of glutamatergic neurons, especially those located in the DGiml [7].

Although its function in the brain is still not well understood, it may play a role in both glutamatergic and dopaminergic neurotransmission [9], [10], [11]. For example, knockdown of endogenous dysbindin with siRNA has been shown to reduce glutamate levels in cultured neurons, suggesting that a decrease in dysbindin levels has synaptic consequences [7], [10]. Furthermore, altered dysbindin-1 expression may contribute to cognitive impairments prominent in schizophrenia, including deficits in attention, memory and executive function [12]–[14]. More recently, “sandy” (sdy−/−) mice, spontaneously occurring dysbindin null mice, have been shown to exhibit a number of behavioral abnormalities associated with reductions in forebrain dopamine transmission [15], [16].

In the present study, we investigated the adult neurogenesis in the dentate gyrus (DG) of the hippocampus in syd−/− mice, in order to further understand the roles of dysbindin-1 in the pathogenesis of major psychosis. We found that sdy−/− mice exhibit significantly reduced rate of neuronal differentiation in the DG at 4 weeks after the progenitor proliferation, although all the other parameters of the neurogenesis examined remained unaltered in sdy−/− mice when compared to those in control mice (sdy+/+). This finding indicates that dysbindin plays a role during the differentiation process of the adult hippocampal neurogenesis.

## Results

### Dysbindin expression

**Figure 1:**
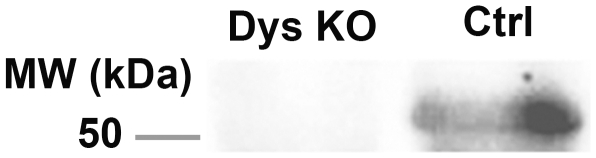
Lack of dysbindin-1 expression in the hippocampus of sdy−/− mice. Homogenates from the hippocampus region of control mice (Ctrl) and sdy−/− mice (Dys KO) were subjected to Western blotting. Primary antibody: a monoclonal mouse dysbindin antibody (1∶1000); secondary antibody: an anti-mouse HRP-linked antibody (1∶2000).

A deletion within the homologous gene in mice accounts for the phenotype known as “sandy” (sdy−/−), which is characterized by albinism and bleeding disorders [17]. This deletion is from nucleotide 3701 of intron 5 to nucleotide 12377 of intron 7, and essentially results in the total loss of dysbindin. We confirmed this lack of dysbindin protein in the sdy−/− mouse hippocampus by Western blot analysis (Figure 1).

### Progenitor proliferation

**Figure 2:**
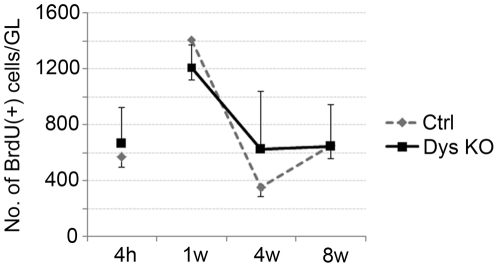
The survival of newborn cells in the SGL of sdy−/− mice. The number of thymidine analogue bromodeoxuridine (BrdU) -incorporated cells was not significantly different in the granular cell layer (GL) between sdy+/+ (Ctrl) and sdy−/− (Dys KO) mice at any time point (4 h, 1 w, 4 w, and 8 w). The data are shown as the mean ± s.d.

To evaluate progenitor cell proliferation in the DG as well as in the entire hippocampus, bromodeoxyuridine (BrdU) was injected intraperitoneally both in sdy−/− and control (sdy+/+) mice (4 months old female mice, n = 4) and the BrdU-labeled cells were counted in mouse hippocampus killed 4 h after the injection. No significant difference was observed between the two groups in terms of the number of BrdU-labeled cells in the DG (sdy+/+ mice, 573±26; sdy−/− mice, 663±87; *p*>0.05) (Figure 2, 4 h), suggesting that BrdU bioavailability was similar in both. The distribution of the dividing cells in the hippocampus of the sdy−/− mice was not obviously different from that seen in sdy+/+ mice. The cells were predominantly found in clusters at the border between the granular layer (GL) and the hilus (HL) (i.e., subgranular layer: SGL) of the DG (Figure S1).

### Progenitor survival

The survival of BrdU-incorporated progenitor cells at 4 months old female mice was also examined in the DG 1 (n = 4), 4 (n = 8) and 8 w (n = 3) after the last BrdU injection. In the SGL, estimates of the total BrdU-positive populations were 1,153±144 (1 w), 625±417 (4 w), and 647±300 (8 w) in sdy−/− mice and 1,282±175 (1 w), 448±69 (4 w), and 652±95 (8 w) in sdy+/+ mice (Figure 2, 1 w, 4 w, 8 w), with no statistically significant differences between groups.

### Progenitor differentiation

**Figure 3:**
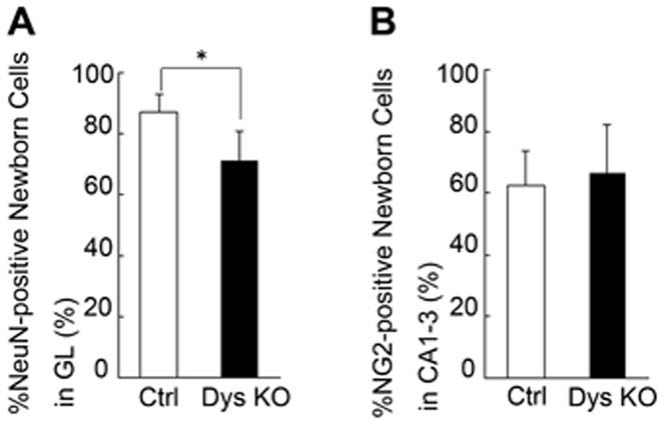
Reduced rate of neuronal differentiation of newborn cells in sdy−/− mice. The rate of NeuN-positive newborn cells in the granular cell layer (GL) of sdy−/− (Dys KO) mice was significantly lower compared to that of sdy+/+ mice (Ctrl) (*p = 0.01), as opposed to that of NG2-positive cells in other areas of the hippocampus (CA1 – 3) at 4 weeks after thymidine analogue bromodeoxuridine (BrdU) injection. The data are shown as the mean ± s.d.

To further examine the effects of dysbindin depletion, differentiation was examined at 4 w after BrdU injection by concurrent immunolabelling for BrdU with neuronal (NeuN) or glial (NG2) markers. NG2 was preferred not only because it serves as a marker for oligodendrocyte progenitor cells (OPCs), but also because OPCs constitute the major dividing glial cell population of the adult CNS [18]. NG2-positive OPCs are distinct from neurons, mature oligodendrocytes, astrocytes, and microglia, and are distributed throughout the gray and white matter [19]. No significant difference was found between groups in either the number or the percentage of BrdU-positive cells that co-labeled for NG2 (Figure S2) in the DG (data not shown); this was also true for other areas of the hippocampus as well (CA1 – 3) (Figure 3).

We found that an equal number of BrdU-positive cells co-labeled with NeuN in the SGL in both the sdy−/− (329±27) and sdy+/+ (326±40) mice 4 w after the last BrdU injection. However, the percentage of BrdU-positive cells that co-labeled with NeuN was significantly less (−15%, *p*<0.01) in sdy−/− mice (71.3±8.3%) than in sdy+/+ mice (87.0±5.3%) (Figure 3), mostly due to the increased, albeit insignificant, number of BrdU-positive cells in the SGL of sdy−/− mice (Figure 2).

## Discussion

Neurogenesis in adult mammalian brains actively occurs at the subventricular zone of the lateral ventricles and at the SGL of DG in the hippocampus. In the present study, we investigated the nature of hippocampal neurogenesis in spontaneously occurring dysbindin null (sdy−/−) mice. We found that, although the numbers of new neurons generated in the SGL of sdy−/− mice are almost equal to those of sdy+/+ mice, the rates of neuronal differentiation stemming from newly generated cells are significantly lower in sdy−/− mice.

**Figure 4:**
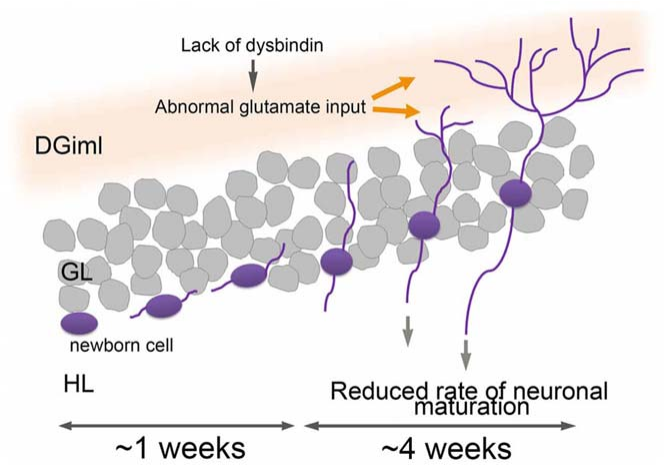
Effect of lack of dysbindin-1 expression on the adult hippocampal neurogenesis. Newborn cells (blue) in the dentate gyrus of sdy−/− (Dys KO) may receive abnormal glutamate inputs within the inner molecular layer (DGiml, orange) due to the lack of dysbindin-1 expression during the critical periods for neuronal differentiation (during the first 3∼4 w from the birth), resulting in the reduced rate of neuronal differentiation. GL and HL are granule cell layer and hilus, respectively.

Newborn cells in the SGL initially receive GABAergic signalling from interneurons within the DG [20]. GABA initially induced the depolarization of immature newborn cells within the first 2–3 w after birth, depending on the gradient of Cl^−^ levels (i.e., intracellular Cl^−^ levels are higher than extracellular ones) [21]–[24]. These cells then gradually migrate into the granular layer and mature as a result of the shift from depolarization to hyperpolarization. Signaling through the NMDA receptor plays a cell autonomous role in the survival of neuronally differentiating newborn cells during the first 3∼4 w after the birth, which coincides with the formation of dendritic spines and functional glutamatergic inputs (Figure 4) [20], [25]–[28].

Although glutamate levels in the entire hippocampal formation of sdy−/− mice remain unaltered compared with those of sdy+/+ mice [15], the expression levels of dysbindin-1 reduced in the DGiml of schizophrenia cases while those of the vesicular glutamate transporter (VGluT-1), a glutamate terminal marker, increased [7], indicating the abnormal glutamate transmission in the DGiml in schizophrenia patients (Figure 4). Moreover, sdy−/− mice exhibited impaired learning in the Morris water maze and T-maze, as well as long-term memory retention deficits in the Barnes circular maze test [15], [16], [29], which is dependent upon a correctly hippocampal functioning and, in part, on the neurogenesis occurring therein [28]. Collectively, our finding that the rate of neuronal differentiation of 4 w-old newborn cells was lower in the DG of sdy−/− mice may explain not only the behavioral abnormalities observed in the sdy−/− mice [15], [16], [29] but also, at least in part, the cognitive, memory or IQ abnormalities associated with genetic variations in human [12]–[14], [30]–[33] and with histopathological abnormalities found in the postmortem brains of schizophrenia patients [5], [7], [8]. Thus, further study of dysbindin-1 genotypes in relation both to specific schizophrenia subtypes and to cognitive endophenotypes are warranted, as does an in-depth investigation of the role of dysbindin in glutamate neurotransmission and in other neuronal functions in the brain.

## Materials and Methods

### Animals

The experiments were largely done on material from a previously published study [15]. “Sandy” (sdy−/−) mice were raised at The Jackson Laboratory, Bar Harbor, ME. Sandy mice have an autosomal recessive coat color mutation that arose spontaneously in the inbred DBA/2J strain. Both sdy−/− mice and wild-type mice derived from heterozygote crossings were used in all experiments. All animals were housed in humidity- and temperature-controlled rooms with a 12-hour (hr) light cycle with free access to food and water. All protocols were approved by the Animal Care and Use Committee of the Tokyo Institute of Psychiatry.

### Western blotting

Following deep anesthetization with sodium pentobarbital, the hippocampus was dissected from each mouse. Hippocampal tissues were homogenated in TNE buffer (20 mM tris-HCl, pH 7.5, 150 mM NaCl, and 1 mM EDTA) containing 1% NP-40 and RIPA buffer and protease inhibitor cocktail (Roche, Sydney, Australia) and centrifuged at 15,000× g for 10 min. Lysates were boiled with SDS sample buffer (0.125 M Tris-HCl, pH 6.8, 10% 2-mercaptoethanol, 4% SDS, 10% sucrose, and 0.004% bromopheonl blue) for 5 min and subjected to SDS–PAGE. Proteins were separated on SDS–PAGE and electrotransferred onto Immobilon-P Transfer Membranes (MILLIPORE, Billerica, USA). Membranes were incubated in PBS containing 5% skim milk and 0.05% Tween 20 for 1 h and blotted with primary antibodies at 4°C overnight. An anti-dysbindin monoclonal antibody (1∶1000) was used as a primary antibody. Mouse monoclonal anti-dysbindin antibody was produced using GST-fused human dysbindin as antigen, as previously described [34], [35]. The membranes were incubated with an anti-mouse HRP-linked secondary antibody (1∶2000, Cell Signalling Technology) for 1 h. Proteins were detected with an ECL kit (Amersham Biosciences, Buckinghamshire, UK) and were then exposed to X-ray films, according to the manufacturer's protocol.

### Bromodeoxyuridine administration

Bromodeoxyuridine (BrdU; Sigma, St. Louis, MO) was dissolved in phosphate buffer saline (PBS), pH 7.4, at a concentration of 10 mg/ml and filter sterilized. Mice were divided into four treatment groups (Group-I ∼ Group-IV). In Group-I, mice received one injection of BrdU (50 µg/g of body weight, i.p.) and were killed 4 h later. In Group-II to -IV, mice received daily injections of BrdU (50 µg/gm of body weight, i.p.) for 3 consecutive days. The mice were then killed 1 (Group-II), 4 (Group-III), or 8 (Group-IV) weeks (w) after the final injection.

### Tissue processing

The animals were anesthetized and transcardially perfused with PBS followed by 4% paraformaldehyde in PBS. Brains were removed, post-fixed for 1 day at 4°C, and then cryoprotected overnight in 20% sucrose in PBS. Coronal sections were cut with a freezing microtome through the hippocampus at 40 µm. Four 1-in-5 series were collected, plated onto glass slides, and stored at −30°C until histochemical analysis.

### BrdU detection

To allow for the detection of BrdU-labeled cells (see below), sections were pretreated for 30 min in 2N HCl at 37°C to denature DNA. The sections were then incubated for 10 min in 100 mM sodium borate, pH 8.5, to neutralize the residual acid.

### Immunofluorescence

Sections were incubated for 30 min in PBS containing 5% goat serum and 0.4% Triton X-100. Using the same buffer solution, the sections were incubated overnight at 4°C in primary antibodies (monoclonal rat anti-BrdU (Harlan Sera-Lab); polyclonal rabbit anti-NG2 (Chemicon, Temecula, CA); monoclonal mouse anti-neuron-specific nuclear protein (NeuN; Chemicon)), followed by 2 h at room temperature in corresponding fluorochrome-conjugated goat secondary antibodies (anti-mouse FITC, anti-mouse rhodamine Red-X (RRX), anti-rabbit Cy5, anti-rabbit FITC, anti-rabbit RRX, and/or anti-rat FITC; all from Jackson ImmunoResearch). Each of the above steps was followed by four 5-min rinses in PBS. The sections were mounted onto gelatin-coated slides, dried, and coverslipped with ProLong antifade medium (Molecular Probes, Eugene, OR).

### Cell counting

Cell counts were performed by an observer who was unaware of the treatment status of the animals. For each animal, a complete series of 1-in-5 sections was analyzed with a light microscope at 600 x magnification. The number of immunoreactive cells was counted in the granular layer (GL) (4 h, 1 w, 4 w, and 8 w) and in the entire hippocampus (4 w). We defined the GL as the granular cell layer plus the areas ∼10 µm deep in the subgranular zone and 5 µm deep in the molecular layer of the DG. The total number of BrdU-positive cells was estimated by multiplying the number of profiles by 5. For cell phenotyping, BrdU-positive cells were analyzed for colocalization with NeuN (in the GL) or NG2 (in the hippocampus). Data were analyzed by ANOVA followed by Scheffé's test for *post hoc* multiple comparisons (Figure 2 and 3). This was done for the two groups, normal controls (sdy+/+) and “Sandy” (sdy−/−) mice.

## Supporting Information

Figure S1 BrdU-positive cells in dentate gyrus (DG) of sdy−/− mice. Sections were the DG of sdy−/− mice after 4 weeks from the last thymidine analogue bromodeoxuridine (BrdU) injections. BrdU-incorporating cells (green) were located mostly between the granular layer (GL) and the hilus (HL). Nuclei were visualized by using Topro-3 (Invitrogen, blue). Images were collected on high-resolution confocal microscopy (LSM510 excitator, Zeiss). Confocal *z* stacks were captured for each section (0.76–0.78 µm increments) using a 20x objective (C-Apochromat, Zeiss). Composite images were reconstructed using Imaris 5.0.3 software (Zeiss). Scale bars represent 100 µm.
(TIF)

Figure S2 NG2-positive oligodendrocyte progenitor cells (OPCs) in the hippocampus of sdy−/− mice. Sections were the hippocampus of sdy−/− mice after 4 weeks from the last thymidine analogue bromodeoxuridine (BrdU) injections. NG2 labeling is shown in red (arrow heads). Arrows indicate BrdU-positive (green), NG2-positive OPCs. Nuclei were visualized by using Topro-3 (Invitrogen, blue). Images were collected on high-resolution confocal microscopy (LSM510 excitator, Zeiss). Confocal *z* stacks were captured for each section (0.36 µm increments) using a 40x water immersion objective (C-Apochromat, Zeiss). Composite images were reconstructed using Imaris 5.0.3 software (Zeiss). Scale bars represent 50 µm.
(TIF)
