# Supplementary figures and images for: Reduced Rate of Neural Differentiation in the Dentate Gyrus of Adult Dysbindin Null (Sandy) Mouse
Source: PLoS One. 2011 Jan 18;6(1):e15886. doi: 10.1371/journal.pone.0015886 (PMC3022736; doi:10.1371/journal.pone.0015886)

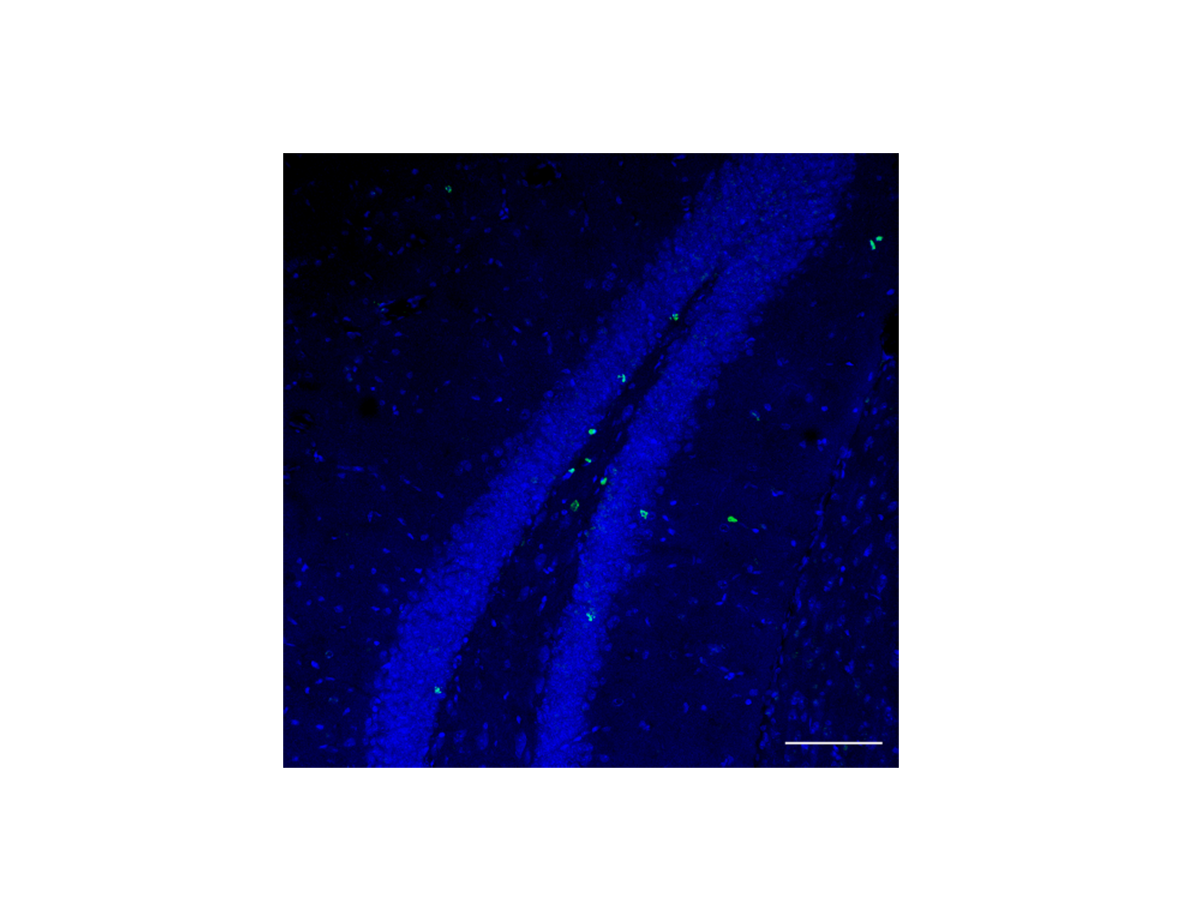

Supplement: Figure S1 — BrdU-positive cells in dentate gyrus (DG) of sdy−/− mice. Sections were the DG of sdy−/− mice after 4 weeks from the last thymidine analogue bromodeoxuridine (BrdU) injections. BrdU-incorporating cells (green) were located mostly between the granular layer (GL) and the hilus (HL). Nuclei were visualized by using Topro-3 (Invitrogen, blue). Images were collected on high-resolution confocal microscopy (LSM510 excitator, Zeiss). Confocal z stacks were captured for each section (0.76–0.78 µm increments) using a 20x objective (C-Apochromat, Zeiss). Composite images were reconstructed using Imaris 5.0.3 software (Zeiss). Scale bars represent 100 µm. (TIF) [file pone.0015886.s001.tif]

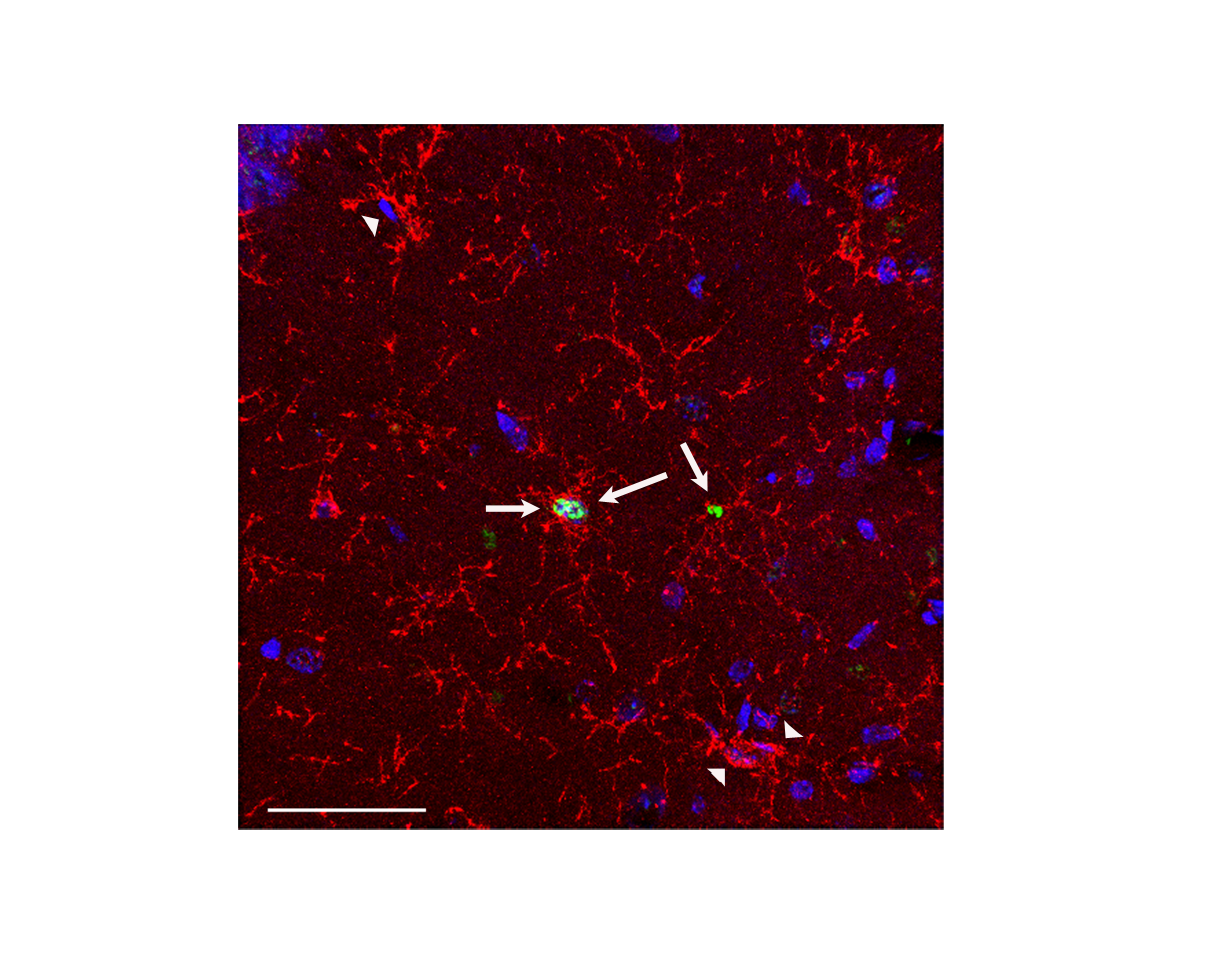

Supplement: Figure S2 — NG2-positive oligodendrocyte progenitor cells (OPCs) in the hippocampus of sdy−/− mice. Sections were the hippocampus of sdy−/− mice after 4 weeks from the last thymidine analogue bromodeoxuridine (BrdU) injections. NG2 labeling is shown in red (arrow heads). Arrows indicate BrdU-positive (green), NG2-positive OPCs. Nuclei were visualized by using Topro-3 (Invitrogen, blue). Images were collected on high-resolution confocal microscopy (LSM510 excitator, Zeiss). Confocal z stacks were captured for each section (0.36 µm increments) using a 40x water immersion objective (C-Apochromat, Zeiss). Composite images were reconstructed using Imaris 5.0.3 software (Zeiss). Scale bars represent 50 µm. (TIF) [file pone.0015886.s002.tif]
